# Tobacco use and its determinants in the 2015 Kenya WHO STEPS survey

**DOI:** 10.1186/s12889-018-6058-5

**Published:** 2018-11-07

**Authors:** Christine Ngaruiya, Hussein Abubakar, Dorcas Kiptui, Ann Kendagor, Melau W Ntakuka, Philip Nyakundi, Gladwell Gathecha

**Affiliations:** 1 Yale School of Medicine, New Haven, CT USA; 2 Division of Non-Communicable Diseases, Ministry of Health, Nairobi, Kenya; 3 Field Epidemiology and Laboratory Training Program, Ministry of Health, Nairobi, Kenya; 4 Alcohol Control Focal Point, Ministry of Health, Nairobi, Kenya

**Keywords:** Noncommunicable disease, Tobacco, Public health, Kenya, Africa

## Abstract

**Background:**

According to the World Health Organization (WHO), in 2015, over 1.1 billion people smoked tobacco, which represents around 15% of the global population. In Africa, around one in five adults smoke tobacco. The 2014 Kenya Global Adult Tobacco Survey reported that 2.5 million adults use tobacco products. The objective of our study was to describe patterns and determinants of tobacco use from the 2015 Kenya STEPS survey, including use of “smokeless” tobacco products and the more novel e-cigarettes.

**Methods:**

The WHO STEPwise approach to surveillance (STEPS) was completed in Kenya between April and June 2015. Logistic regression analyses was used to assess factors affecting prevalence and frequency of tobacco use. Sociodemographic variables associated with tobacco use were considered: age, sex, level of education, wealth quintile, and residence. The relationship with alcohol as an intervening risk factor was also assessed. Our main outcomes of interest were current tobacco use, daily tobacco use and use of smokeless tobacco products.

**Results:**

Of 4484 respondents, 605 (13.5%) reported being current tobacco users. Most active tobacco users were male (*n* = 507/605, 83.8%). Three out of four tobacco users (*n* = 468/605, 77.4%) reported being less than 50 years old, with the average start age being 21 (20.6, 95% CI 19.3–21.8) and the average quit age 27 (27.2, 95% CI 25.8–28.6). Most tobacco users had only ever attended up to primary school (*n* = 434/605, 71.7%). Men had nearly seven times higher odds of being tobacco users as compared to women (OR 7.63, 95% CI 5.63–10.33). Alcohol use had a positive effect on tobacco use. Finally, less than ten respondents reported having used e-cigarettes.

**Conclusion:**

The 2015 Kenya WHO STEPS provided primary data on the status of tobacco use in the country and other leading NCD risk factors, such as alcohol, and associated diseases. Our findings highlight key target populations for tobacco cessation efforts: young people, men, those with lower levels of education, and alcohol consumers. Further data is needed on the use of smokeless tobacco, and its impact on smoked tobacco products, as well as on the novel use of e-cigarettes.

## Background

Tobacco use and exposure is one of the leading risk factors for non-communicable diseases (NCDs), a major public health threat globally and a preventable cause of morbidity and mortality [1–10]. According to the World Health Organization (WHO), in 2015, over 1.1 billion people smoked tobacco, which represents around 15% of the global population [2]. Smoking was attributable to 11.5% of total global deaths in 2015 and 150 million disability-adjusted life years [10]. Nearly 80% of these individuals live in low- and middle-income countries which constitutes the majority of countries in the African region [3, 8]. Use of tobacco products and exposure to tobacco smoke kills around 6 to 7 million people each year [1, 2, 5]. Over 5 million of the deaths result from direct tobacco use and a further 600, 000 result from non-smokers being exposed to second-hand smoke [1, 9].

Although Africa has had low prevalence of tobacco use [11, 12], projections show increase with population growth [9, 13]. Around one in five adults in the region smokes tobacco [3, 9, 10, 14–16], with the highest rates in Sierra Leone (37.7%) and lowest in Sao Tome & Principe (6.75%) [15]. Among users, there is a disproportionally higher proportion among men as compared to women, at 21 and 3%, respectively. Eighteen percent of young people (13–15 years old) in Africa currently use some form of tobacco product [3, 17]. Studies show use of tobacco as early as under 7 years of age in western Africa [18] and in Kenyan secondary schools as early as 11 years old [19]. The landscape of tobacco use in Africa is changing with increasing use of alternative forms of tobacco being used such as e-cigarettes including use by women [10, 20–23].

According to the 2014 Kenya Global Adult Tobacco Survey (GATS), a nationally representative household survey as a component of global tobacco surveillance system, 2.5 million adults use tobacco products in Kenya, which constitutes 11.6% of the population [24]. The 2014 Kenya Demographic and Health Survey (KDHS) estimated 16% of men and 0.4% of women aged 15–45 use cigarettes [25]. In its 2014 global NCD report, the WHO estimated current tobacco use at 13% [26]. These figures are consistent with findings by Gathecha et al. in a review of previous studies on tobacco use in Kenya that demonstrated prevalence range between 3.8 and 19% [27]. The Kenya GATS showed there was a higher proportion of men that use tobacco products as compared to women at 19.1 and 4.5%, respectively, and that around 6% of users smoke tobacco daily while 1.8% report not to use daily [24]. Alcohol has been shown to be associated with increased tobacco use in sub-populations such as rural Kenya and Nairobi slums [28, 29]. The GATS Kenya survey uses the definition of smokeless tobacco products as those wholly or partly made from tobacco and do not need to be ignited for it to be consumed.

Kenya ratified the WHO Framework Convention for Tobacco Control (FCTC) in 2004, and implemented the Kenya Tobacco Control Act in 2007 [2, 6, 30]. The country has an agency dedicated to tobacco control, and attained the highest level of achievement for tobacco advertising, promotion and sponsorship deterrent efforts, which are both measures of success according to the WHO FCTC and MPOWER [31–34]. While gains have been made, given these guidelines, there is still need for greater attention, especially first establishing the levels and determinants of tobacco use [6, 8, 27, 35, 36].

The WHO STEPwise approach to surveillance of NCD risk factors (STEPS) survey is part of a global surveillance strategy in response to the growing need for data on country level trends in NCDs and injuries and the related risk factors [37]. The Kenya STEPs survey is the first nationally representative survey to collect comprehensive information on risk factors for NCDs and injuries [38]. The survey serves as an evidence base to strengthen NCD prevention and control initiatives in the country. The objective of our study was to describe patterns and determinants of tobacco use in Kenya, including current and former use. We also assessed the influence of alcohol use amongst tobacco users in a national sample, a novel addition to the literature. Finally, we examined the more novel use of e-cigarettes.

## Methods

The WHO STEPwise approach to surveillance (WHO STEPS) study was completed in Kenya between April and June 2015 [38]. The STEPS instrument is a cross-culturally validated survey tool used to assess burden of leading NCDs and associated lifestyle risk factors in a nationally representative sample. A cross-sectional household survey approach was used, with a three-stage cluster sample design involving selection of clusters from the National Bureau of Statistics household-based sampling frame (NASSEP V), households and eligible individuals as further described in the Kenya WHO STEPS report [38]. The individual identified as the head of household at the time of contact responded to the survey. Written informed consent was obtained from the selected individual. Criteria for inclusion was individuals aged 18–69 years old. Those that refused consent were excluded from the study.

### Materials

The STEPS tool was used to collect data electronically on IPAQ Personal Digital Assistants (PDAs). Respondents completed a verbal component of the survey, and physical and biochemical measurements were also collected in line with the STEPS approach [37]. Additional survey questions on tobacco use which are region-specific, such as on smokeless tobacco products are from the Global Adult Tobacco Survey (GATS), which is another validated tool [39]. All analyses were done using Stata 14 (Stata corporation, College Station, TX).

### Independent variables

The sociodemographic factors age, sex, number of years of education, occupation, wealth quintile, residence and alcohol consumption were considered as independent variables. The sociodemographic variables selected are well known in the literature to have association with tobacco use [39]. Wealth quintile is presented in five quintiles. The wealth index was generated using the multivariate statistical technique (Principal Components Analysis), according to methodology outlined by the DHS (Demographic and Health Surveys) program [25]. Principal components are weighted averages of the variables used to construct them. Among all weighted averages, the first principal component is usually the one that has the greatest ability to predict the individual variables that make it up, where prediction is measured by the variance of the index. Variables included in wealth index determination were: type of dwelling, ownership of the dwelling, construction materials of the dwelling, source of cooking fuel, source of lighting fuel, household possessions/ goods, source of water for household consumption, and type of sanitation facility, as indicated by the Kenya STEPS report [38]. Alcohol consumption is also an established independent risk factor for tobacco use [40, 41].

### Dependent variables

Our main outcomes of interest for the study were to assess determinants of daily tobacco use and smokeless tobacco use in Kenya. For bivariate analysis, the distribution of tobacco use by sociodemographic status was also assessed for those that have never used tobacco, all current tobacco users (both smoked and smokeless tobacco products), and those that have used e-cigarettes. The distribution of tobacco use amongst alcohol users was also assessed in bivariate analysis.

### Descriptive analysis

For descriptive analyses, frequency and proportions are presented. Percentages presented were weighted for the population.

### Multivariable analysis

All sociodemographic variables were included in final regression models using a stepwise process with inclusion in the final model if found to be statistically significant. This was true except for occupation given the original coding of the variable in the survey that was not felt to be meaningful for our study. The variable sex was maintained in the three models, even though it was only found to have a statistically significant relationship with daily tobacco use given the hypothesized importance of the role of sex on tobacco use.

The variable, alcohol consumption, was hypothesized to be an interaction factor given the known association with aforementioned sociodemographic factors and tobacco use. It was therefore felt to be along the causal pathway, and treated as such. Where there was statistical evidence for interaction once testing was performed, this is discussed in the text. Likelihood ratio testing was used to assess for interaction. Table findings present unadjusted and adjusted odds ratios for variables included in the final model. For those variables that were not included in the final model, only the unadjusted odds ratio are presented. Finally, both unadjusted and adjusted odds ratios are presented with associated 95% confidence intervals (CIs).

## Results

There were 4484 respondents in our analysis, and 605 (13.5%) of them reported being current tobacco users. The vast majority of tobacco users were smokers. Most active tobacco users were male (*n* = 507/605, 83.8%) as shown in Table 1. Three out of four tobacco users (*n* = 468/605, 77.4%) reported being less than 50 years old, with the average start age being 21 (20.6, 95% CI 19.3–21.8) and the average quit age 27 (27.2, 95% CI 25.8–28.6). Another 353 respondents or 8% of the population were former tobacco users. Only 4% of the population reported using smokeless tobacco products, but most of those using smokeless tobacco do so exclusively, as shown in Table 2. Less than 1% of the population reported using both smoked and smokeless tobacco products. Finally, less than ten actual respondents reported ever having used e-cigarettes.

**Table 1:** Breakdown by sociodemographic status across current and former tobacco usage

Characteristic	Ever used tobacco	Currently smoke tobacco	Currently use smokeless tobacco	Ever used electronic cigarette	Former smoker	Currently using tobacco	Former tobacco users	Daily use tobacco
	Yes (*n*, (%))	Yes (*n*, (%))	Yes (*n*, (%))	Yes (*n*, (%))	Yes (*n*, (%))	Yes (*n*, (%))	Yes (*n*, (%))	Yes (*n*, (%))
Sex								
Male	812 (37.1)	435 (19.9)	88 (4.0)	6 (0.3)	290 (16.3)	507 (23.2)	301 (13.8)	406 (18.6)
Female	145 (6.3)	21 (0.9)	75 (3.3)	1 (0.1)	39 (1.7)	93 (4.1)	52 (2.3)	71 (3.1)
Age groups								
18–29	284 (13.8)	152 (7.4)	56 (2.7)	2 (0.1)	74 (3.8)	200 (9.7)	84 (4.1)	139 (6.7)
30–39	245 (23.4)	132 (12.6)	31 (3.0)	4 (0.4)	77 (8.3)	156 (14.9)	86 (8.3)	119 (11.4)
40–49	200 (28.8)	89 (12.8)	26 (3.7)	0 (0.0)	87 (14.1)	112 (16.2)	88 (12.6)	100 (14.4)
50–59	137 (30.8)	51 (11.6)	29 (6.6)	1 (0.1)	53 (13.4)	80 (18.1)	56 (12.6)	75 (16.9)
60–69	91 (38.1)	33 (13.7)	21 (8.9)	0 (0.0)	37 (17.8)	52 (21.7)	39 (16.5)	44 (18.4)
Education level								
No formal education	139 (24.7)	33 (5.8)	86 (15.4)	0 (0.0)	21 (3.9)	113 (20.1)	26 (4.6)	91 (16.2)
Primary education	501 (24.5)	282 (13.8)	48 (2.3)	2 (0.1)	167 (9.3)	321 (15.7)	180 (8.8)	266 (13.0)
Secondary and above	316 (16.9)	142 (7.6)	29 (1.5)	6 (0.3)	140 (7.9)	171 (9.1)	147 (7.8)	119 (6.3)
Residence								
Rural	598 (21.5)	256 (9.2)	130 (4.7)	3 (0.1)	208 (8.1)	373 (13.4)	223 (8.0)	306 (11.0)
Urban	359 (21.0)	201 (11.8)	32 (1.9)	5 (0.3)	121 (7.9)	228 (13.3)	130 (7.6)	171 (10.0)
Occupation								
Unemployed	288 (16.0)	125 (6.9)	94 (5.2)	2 (0.1)	62 (3.7)	216 (12.0)	72 (4.0)	176 (9.9)
Employed	669 (24.9)	332 (12.4)	69 (2.6)	6 (0.2)	266 (11.1)	389 (14.5)	281 (10.5)	300 (11.1)
Ever consumed alcohol								
No	181 (7.1)	73 (2.9)	58 (2.3)	0 (0.0)	45 (1.8)	128 (5.0)	53 (2.1)	108 (4.2)
Yes	775 (40.1)	383 (19.8)	105 (5.4)	7 (0.4)	284 (18.0)	473 (24.4)	298 (15.4)	369 (19.1)
Wealth band								
Poorest	198 (23.3)	75 (8.9)	84 (9.9)	1 (0.1)	39 (4.9)	155 (18.3)	42 (5.0)	123 (14.5)
Second	200 (21.4)	97 (10.4)	31 (3.3)	0 (0.0)	71 (8.4)	124 (13.3)	76 (8.1)	107 (11.5)
Middle	186 (22.7)	95 (11.6)	19 (2.3)	2 (0.2)	68 (9.3)	112 (13.7)	74 (9.1)	94 (11.4)
Fourth	201 (24.2)	101 (12.1)	8 (0.9)	1 (0.1)	87 (11.7)	106 (12.8)	95 (11.4)	89 (10.7)
Richest	171 (16.3)	88 (8.4)	21 (2.0)	3 (0.3)	63 (6.5)	108 (10.3)	65 (6.2)	64 (6.1)
Marital status								
Not married	201 (19.3)	119 (11.4)	20 (1.9)	3 (0.3)	64 (6.8)	133 (12.8)	68 (6.6)	96 (9.2)
Married	594 (20.2)	257 (8.8)	97 (3.3)	4 (0.2)	226 (8.3)	350 (11.9)	244 (8.3)	295 (10.0)
Formerly married	161 (31.9)	80 (15.9)	45 (8.9)	0 (0)	38 (8.8)	122 (24.1)	40 (8)	86 (17.0)
*Total*^a^	*956 (21.3)*	*456 (10.2)*	*163 (3.6)*	*7 (0.2)*	*328 (8)*	*605 (13.5)*	*353 (7.9)*	*477 (10.6)*

Key: ^a^Summation by column within a characteristic may not equal the total due to weighting done on the data and rounding off to whole numbers

**Table 2:** Breakdown by sociodemographic status across forms of tobacco used (smoked and smokeless)

Characteristic	Non user	One form only	Smoked & smokeless	Total (*N*)
	*n*, (%)	*n*, (%)	*n*, (%)	
Sex				
Male	1675 (76.6)	493 (22.6)	16 (0.7)	2186
Female	2203 (95.9)	92 (4.0)	3 (0.1)	2298
Total*	*3879 (86.5)*	*585 (13.1)*	*18 (0.4)*	*4484*
Age groups				
18–29	1860 (90.2)	194 (9.4)	7 (0.3)	2062
30–39	886 (84.8)	150 (14.4)	7 (0.6)	1045
40–49	582 (83.8)	110 (15.9)	2 (0.3)	695
50–59	362 (81.8)	81 (18.2)	0 (0.0)	443
60–69	188 (78.4)	49 (20.7)	2 (1.0)	239
Total*	*3879 (86.5)*	*585 (13.1)*	*18 (0.4)*	*4484*
Education level				
No formal education	450 (79.9)	107 (19)	6 (1.1)	563
Primary education	1722 (84.3)	313 (15.3)	8 (0.4)	2043
Secondary and above	1706 (90.9)	166 (8.8)	4 (0.2)	1877
Total*	*3879 (86.5)*	*585 (13.1)*	*18 (0.4)*	*4484*
Residence				
Rural	2402 (86.5)	360 (13.0)	13 (0.5)	2776
Urban	1477 (86.5)	225 (13.2)	5 (0.3)	1708
Total*	*3879 (86.5)*	*585 (13.1)*	*18 (0.4)*	*4484*
Occupation				
Unemployed	1583 (88)	211 (11.7)	5 (0.3)	1799
Employed	2295 (85.5)	374 (13.9)	14 (0.5)	2685
Total*	*3879 (86.5)*	*585 (13.1)*	*18 (0.4)*	*4484*
Ever consumed alcohol				
No	2421 (95.0)	125 (4.9)	3 (0.1)	2549
Yes	1457 (75.3)	460 (23.8)	15 (0.8)	1934
Total*	*3877 (86.5)*	*585 (13.1)*	*18 (0.4)*	*4483*
Wealth band				
Poorest	693 (81.7)	150 (17.7)	4 (0.5)	848
Second	813 (86.7)	120 (12.8)	4 (0.5)	937
Middle	707 (86.3)	108 (13.2)	3 (0.4)	818
Fourth	725 (87.2)	104 (12.5)	2 (0.2)	832
Richest	941 (89.8)	103 (9.8)	4 (0.4)	1049
Total*	*3879 (86.5)*	*585 (13.1)*	*18 (0.4)*	*4484*
Marital status				
Not married	906 (87.2)	124 (11.9)	8 (0.8)	1039 (100)
Married	2588 (88.1)	342 (11.7)	7 (0.3)	2938 (100)
Formerly married	385 (75.9)	119 (23.5)	3 (0.6)	507 (100)
Total*	*3879 (86.5)*	*585 (13.1)*	*18 (0.4)*	*4484*

Key: *Summation by column within a characteristic may not equal the total due to weighting done on the data and rounding off to whole numbers

Most tobacco users had only ever attended up to primary school education (*n* = 434/605, 71.7%). Conversely, only 1.2% of current tobacco users had completed university education or higher (data not shown in table). The proportion of tobacco users in rural and urban areas are similar (21.5 and 21%, respectively), however, there is a predominance of tobacco smoking in urban areas and a predominance of smokeless tobacco use in rural areas. Of the seven respondents who reported e-cigarette use, most were male, younger than 40 years old, had attained some education, and lived in urban areas.

There was evidence of a statistically significant association with current tobacco use and sex, age group, level of education, occupation, wealth index, marital status, alcohol use, and heavy episodic drinking, as shown in Table 3. Males, those that were older, those with less education, those that are employed, those that are formerly married, and those with history of alcohol use were found to have higher odds of current tobacco use than their counterparts, respectively. There was no statistically significant evidence of an association with residence, however, after controlling for confounders, those in the rural area were found to have a lower odds of tobacco use (OR 0.65, 95% CI 0.49–0.86).

**Table 3:** Covariates associated with current tobacco use in Kenya

Current tobacco use	Crude Odds Ratio^a^		Adjusted Odds Ratio^a^	
	OR (95% CI)	*p*-value	OR (95% CI)	*p*-value
Sex				
Female	1.00		1.00	
Male	7.11 (5.65, 8.93)	0.000	7.63 (5.63, 10.33)	< 0.001
Age group				
18–29	1.00		1.00	
30–39	1.65 (1.32, 2.06)	0.000	1.33 (0.91, 1.94)	0.137
40–49	1.78 (1.39, 2.28)	0.000	1.13 (0.74, 1.72)	0.585
50–59	2.06 (1.55, 2.72)	0.000	1.76 (1.14, 2.74)	0.011
60–69	2.55 (1.81, 3.58)	0.000	0.81 (0.45, 1.46)	0.481
Education level				
No formal education	1.00		1.00	
Primary complete	0.74 (0.59, 0.94)	0.015	0.29 (0.20, 0.43)	< 0.001
Secondary and above	0.40 (0.31, 0.52)	0.000	0.11 (0.07, 0.17)	< 0.001
Residence				
Urban	1.00		1.00	
Rural	1.00 (0.84, 1.19)	0.971	0.65 (0.49, 0.86)	0.003
Occupation				
Unemployed	1.00		1.00	
Employed	1.22 (1.02, 1.46)	0.029	0.67 (0.52, 0.85)	0.001
Ever used alcohol				
No	1.00		1.00	
Yes	6.17 (5.03, 7.58)	0.000	3.36 (2.52, 4.48)	< 0.001
Episodic alcohol drinking				
No alcohol	1.00		1.00	
Binge drinking	8.75 (7.18, 10.65)	0.000	1.36 (0.56, 3.34)	0.499
Non-heavy drinking	2.82 (1.97, 4.04)	0.000	0.28 (0.03, 2.38)	0.246
Wealth band				
Poorest	1.00		1.00	
Second	0.68 (0.53, 0.88)	0.003	0.68 (0.45, 1.04)	0.072
Middle	0.71 (0.54, 0.92)	0.010	0.58 (0.37, 0.91)	0.019
Fourth	0.65 (0.50, 0.86)	0.002	0.61 (0.38, 0.97)	0.037
Richest	0.51 (0.39, 0.66)	0.000	0.63 (0.38, 1.06)	0.082
Marital status				
Not married	1.00		1.00	
Married	0.92 (0.75, 1.14)	0.469	0.69 (0.51, 0.93)	0.015
Formerly married	2.17 (1.65, 2.85)	0.000	2.10 (1.41, 3.11)	< 0.001

Key: ^a^All sociodemographic variables (except occupation) were included in final regression models if found to be statistically significant. This was true except for occupation given the original coding of the variable in the survey that was not felt to be meaningful for our study. The variable sex was maintained in the three models, even though it was only found to have a statistically significant relationship with daily tobacco use given the hypothesized importance of the role of sex on tobacco use

Men had nearly seven times higher odds of being tobacco users as compared to women (OR 7.63, 95% CI 5.63–10.33), see Table 3. After controlling for confounding, the strength of the relationship between age and tobacco use was less evident, however a trend showing an increase in use with age still remained, and those in the 50–59 year age group, were nearly twice as likely as compared to their younger counterparts aged 18–29 to be engaged in tobacco use (OR 1.76, 95% CI 1.14–2.74). Those with at least primary education were less likely to be currently using tobacco. Those that use alcohol have three times higher odds of being current smokers (OR 3.36, 95% CI 2.52–4.48). Of note, after controlling for confounding the relationship between tobacco use and heavy episodic drinking, in particular, was no longer evident also as shown in Table 3.

### Daily tobacco use

The majority of tobacco users reported doing so daily (*n* = 477/605, 78.8%), as shown in Table 1. Nine out of ten smokers reported this also (85%, *n* = 406/477). Those that reported ever having consumed alcohol, were also more commonly daily tobacco users (*n* = 369/477, 77.3%). There were similar findings of association for daily tobacco use (Table 4) as compared to current tobacco use (Table 3) by sociodemographic status. Two main differences were a stronger association with age (despite a smaller sample size), with those being in the 50–59 year age group having around three times higher odds than their younger counterparts with daily tobacco use. There was a slightly weaker association with alcohol use for those using tobacco daily, and marital status was no longer a determinant for daily tobacco use, after controlling for confounding. There was no statistical evidence of interaction with alcohol use affecting the outcome daily tobacco use.

**Table 4:** Covariates associated with daily tobacco use in Kenya

Daily tobacco use	Crude Odds Ratio^a^		Adjusted Odds Ratio^a^	
	OR (95% CI)	*p*-value	OR (95% CI)	*p*-value
Sex				
Female	1.00		1.00	
Male	7.16 (5.52, 9.28)	< 0.001	7.48 (5.34, 10.48)	< 0.001
Age group				
18–29	1.00		1.00	
30–39	1.79 (1.38, 2.31)	< 0.001	1.35 (0.87, 2.08)	0.181
40–49	2.33 (1.77, 3.06)	< 0.001	1.39 (0.86, 2.23)	0.176
50–59	2.81 (2.08, 3.81)	< 0.001	2.57 (1.61, 4.11)	< 0.001
60–69	3.12 (2.16, 4.52)	< 0.001	1.36 (0.74, 2.51)	0.324
Education level				
No formal education	1.00		1.00	
Primary complete	0.77 (0.60, 1.00)	0.050	0.28 (0.18, 0.43)	< 0.001
Secondary and above	0.35 (0.26, 0.47)	< 0.001	0.12 (0.07, 0.20)	< 0.001
Residence				
Urban	1.00		1.00	
Rural	1.11 (0.91, 1.36)	0.288	0.63 (0.46, 0.85)	0.002
Occupation				
Unemployed	1.00		1.00	
Employed	1.14 (0.93, 1.38)	0.200	0.58 (0.45, 0.76)	< 0.001
Ever used alcohol				
No	1.00		1.00	
Yes	5.35 (4.28, 6.69)	< 0.001	2.54 (1.85, 3.49)	< 0.001
Episodic alcohol drinking				
No alcohol	1.00		1.00	
Binge drinking	8.03 (6.49, 9.93)	< 0.001	0.77 (0.30, 1.96)	0.588
Non-heavy drinking	3.41 (2.34, 4.97)	< 0.001	0.52 (0.06, 4.59)	0.560
Wealth band				
Poorest	1.00		1.00	
Second	0.76 (0.58, 1.00)	0.054	0.91 (0.58, 1.41)	0.665
Middle	0.76 (0.57, 1.01)	0.061	0.56 (0.33, 0.93)	0.026
Fourth	0.70 (0.53, 0.94)	0.018	0.69 (0.41, 1.15)	0.150
Richest	0.38 (0.28, 0.52)	< 0.001	0.47 (0.26, 0.86)	0.014
Marital status				
Not married	1.00		1.00	
Married	1.10 (0.87, 1.41)	0.429	0.73 (0.53, 1.02)	0.066
Formerly married	2.03 (1.48, 2.77)	< 0.001	1.41 (0.91, 2.17)	0.120

Key: ^a^All sociodemographic variables (except occupation) were included in final regression models if found to be statistically significant. This was true except for occupation given the original coding of the variable in the survey that was not felt to be meaningful for our study. The variable sex was maintained in the three models, even though it was only found to have a statistically significant relationship with daily tobacco use given the hypothesized importance of the role of sex on tobacco use

### Smokeless tobacco use

Smokeless tobacco use was fairly evenly distributed across sex, with a slight increased prevalence of use amongst males (4%, as compared to 3.3% of females) Table 1. Around three quarters of smokeless tobacco users were in the poorest wealth quintile.

However, of all sociodemographic variables, only education level and occupation were found to have a statistically significant association with using smokeless tobacco after controlling for confounding. An increase in the number of years of education was found to be protective. In respondents with at least a high school education, they had 100 times lower odds of using smokeless tobacco as compared to those that had never attended school (OR < 0.01, 95% CI 0–0.09), as shown in Table 5.

**Table 5:** Covariates associated with smokeless tobacco use in Kenya

Smokeless tobacco use	Crude Odds Ratio^a^		Adjusted Odds Ratio^a^	
	OR (95% CI)	*p*-value	OR (95% CI)	*p*-value
Sex				
Female	1.00		1.00	
Male	1.23 (0.90, 1.69)	0.189	1.51 (0.95, 2.41)	0.079
Age group				
18–29	1.00		1.00	
30–39	1.11 (0.71, 1.74)	0.635	0.94 (0.47, 1.87)	0.854
40–49	1.38 (0.85, 2.22)	0.190	2.04 (1.01, 4.11)	0.047
50–59	2.53 (1.59, 4.01)	< 0.001	1.99 (0.98, 4.03)	0.057
60–69	3.54 (2.11, 5.94)	< 0.001	1.1 (0.46, 2.66)	0.826
Education level				
No formal education	1.00		1.00	
Primary complete	0.13 (0.09, 0.19)	< 0.001	0.12 (0.06, 0.22)	< 0.001
Secondary and above	0.09 (0.06, 0.13)	< 0.001	0.01 (0, 0.09)	< 0.001
Residence				
Urban	1.00		1.00	
Rural	2.55 (1.73, 3.77)	< 0.001	1.38 (0.76, 2.52)	0.292
Occupation				
Unemployed	1.00		1.00	
Employed	0.47 (0.34, 0.64)	< 0.001	0.58 (0.39, 0.88)	0.009
Ever used alcohol				
No	1.00		1.00	
Yes	2.49 (1.79, 3.45)	< 0.001	2.58 (1.47, 4.54)	0.001
Episodic alcohol drinking				
No alcohol	1.00		1.00	
Binge drinking	5.19 (3.75, 7.19)	< 0.001	4.84 (1.55, 15.15)	0.007
Non-heavy drinking	1.04 (0.42, 2.55)	0.932	3.52 (0.22, 57.21)	0.377
Wealth band				
Poorest	1.00		1.00	
Second	0.31 (0.21, 0.48)	< 0.001	0.44 (0.22, 0.91)	0.026
Middle	0.21 (0.13, 0.35)	< 0.001	0.56 (0.28, 1.15)	0.117
Fourth	0.09 (0.04, 0.18)	< 0.001	0.04 (0, 0.57)	0.017
Richest	0.19 (0.11, 0.3)	< 0.001	0.16 (0.02, 1.08)	0.060
Marital status				
Not married	1.00		1.00	
Married	1.73 (1.07, 2.82)	0.026	1.21 (0.67, 2.2)	0.522
Formerly married	4.96 (2.9, 8.48)	< 0.001	2.48 (1.27, 4.83)	0.007

Key: ^a^All sociodemographic variables (except occupation) were included in final regression models if found to be statistically significant. This was true except for occupation given the original coding of the variable in the survey that was not felt to be meaningful for our study. The variable sex was maintained in the three models, even though it was only found to have a statistically significant relationship with daily tobacco use given the hypothesized importance of the role of sex on tobacco use

There was also statistical evidence for interactions indicating that the association of age with use of smokeless tobacco is dependent on whether or not respondents engage in heavy episodic drinking. When comparing across sub-groups, those that reported heavy episodic drinking had higher odds of smokeless tobacco use, with up to 26 times higher odds amongst the oldest age group when compared to younger non-consumers of alcohol (OR 25.6, 95% CI 11.0–59.7). For the two lowest wealth bands, those that are binge drinkers also have a higher odds of smokeless tobacco use (OR 2.91, 95% CI 1.69–5 in the lowest, and OR 2.12, 95% CI 1.19–3.77 in the second lowest, respectively).

## Discussion

This study provides the first nationally representative estimates on the prevalence of tobacco use alongside other leading risk factors for NCDs, and disease outcomes related to these risk factors. Overall, 13.5% of the respondents currently use tobacco. This is slightly higher than what was has been reported in the Kenya GATS conducted in 2014 at 11.6% [25], and in some previous studies in the country as shown by Gathecha et al. [27]. The prevalence of tobacco use was also higher in the STEPS as compared to the 2015 GBD study, where for example daily tobacco use for males was 18.6% compared to 14.9%, respectively [10]. The difference between the STEPS survey and GATS could be attributed to the different age ranges of the population sampled. The Kenya GATS examined respondents aged 15 years and above, which may include an age group (15–18 years) that have not yet engaged in tobacco use therefore lowering the prevalence estimate. Alternatively, differences in the STEPS and GATS could be explained by sampling approach. While both the STEPS and GATS use sample frames that originate from the Kenya National Bureau of Statistics (KNBS), cluster selection, and randomization for participant selection differed [25, 38].

The high prevalence found in our study is of concern, and calls for urgent action as tobacco use is not only a major risk factor for health, it also is a major contributor to inequities in health and social development [42–44]. Prevalence levels are higher than in 14 of 30 other sub-Saharan African countries [15], and in other countries in the East African region such as Ethiopia (4.2%) and Uganda (9.6%). It is comparable to Rwanda (12.8%) and Tanzania (14.1%) [45–48]. It is therefore a call for renewed diligent implementation of the WHO FCTC and the Tobacco Control Act (TCA) by all stakeholders [3].

A national tobacco cessation quit line, nicotine replacement therapy and pharmacologic aids for cessation such as Bupropion are available. However, the availability of treatment for tobacco cessation is only at certain sites. Furthermore, coverage for treatment is not guaranteed. Finally, the enforcement of smoke-free zones is still lacking and no current monitoring for effectiveness of policies exists to date [27, 31, 35, 49]. Additionally, more can be done at the healthcare provider to level to educate patients on smoking cessation [50, 51]. Lack of understanding amongst the general population on the effects of tobacco use on chronic disease, such as cancer, continues to be a problem [27, 52].

The changing trend of tobacco use in Africa is also important to highlight. The Africa region is estimated to have the highest predicted increase in tobacco consumption between 2010 and 2025 [53, 54]. This is because the tobacco industry is turning its focus on the youth and especially in Africa which is at the same time experiencing large population increases [10, 12].

Consistent with studies done in other countries in sub-Saharan Africa [15, 16] and other LMICs [55], tobacco use among men is significantly higher than women (OR 7.11 CI 5.65, 8.93). Studies suggest that this phenomenon may be attributable to the tobacco industry consistently targeting men by portraying smoking as a masculine activity with advantages such as increased sexual prowess as well as the community perception towards smoking that is generally more tolerant of male smokers over female smokers [56–59]. Recognizing sex differences in tobacco use is important because it guides which tobacco control interventions should be prioritized in order to reach the group that is most affected [60]. Studies have shown that there should be a difference in interventions such as tobacco messaging and cessation depending on the sex targeted [60–63].

Our study found that majority of the smokers were in the age group 18–29 years and the mean age of initiation was early twenties. This is a vulnerable population that are being targeted by the tobacco industry especially in the African region [17, 20, 54, 64, 65]. The high prevalence of tobacco use among the younger population has been proposed to be driven by the high exposure to advertising, among other factors. For example, 59% of youth are currently being exposed to pro-cigarette billboard advertisements and 15% of these youth in the region are likely to initiate smoking [66]. Addressing tobacco use at this age group is critical as it will determine the future burden of tobacco use and its consequences. This calls for strengthened enforcement of the FCTC and TCA that already have provision on the banning of tobacco advertising, promotion and sponsorship.

In our study, 3.6% of the respondents reported consuming smokeless tobacco while the figure was 4.5% in the GATS [25] and 2.05% for men in the KDHS [15]. The use of smokeless tobacco may be influenced by smoking bans in public places, introduced in Kenya in 2007. Researchers have found that smokeless tobacco use is perceived to be less harmful than smoked tobacco products when these products cause nicotine addiction, cancers and other non-communicable diseases [67]. In our population, higher levels of education were protective against smokeless tobacco use. Educational interventions specific to harmful risks of smokeless tobacco use may have a significant role. There was a higher likelihood of smokeless tobacco use among rural residents than urban residents necessitating the need for intensified campaigns against smokeless tobacco in rural areas.

Alcohol use had a positive effect on current and daily tobacco use. This relationship was true for ever users of tobacco, binge drinkers and non-binge drinkers. This finding is corroborated in literature [41, 65]. The association between tobacco and alcohol use has been explained by many factors including genetics, neurobiological mechanisms, conditioning mechanisms and psychosocial factors [67, 68]. Our study found higher odds among binge drinkers (OR 8.7) than non-binge drinkers (OR 2.8) when exploring current tobacco use. There is, therefore, an added advantage in tailoring messaging to the public against joint tobacco and alcohol use [69, 70]. Additionally, it is prudent for stakeholders working in alcohol control and tobacco control to work collaboratively to maximize impact.

Respondents with no formal education were more likely to be tobacco users. Similar findings are prevalent in other LMICs comparable to Kenya [10, 15, 16, 55]. This has an implication on tobacco control public education. The Ministry of Health and other stakeholders should ensure that they employ a mix of communication channels when distributing anti-tobacco messages. Innovative, culturally appropriate and carefully designed messaging is critical to reaching at-risk groups including adolescents and those with limited ability to read [70–72]. In regards to health warnings on cigarette packaging, the Ministry of Health recently passed regulations for the implementation of graphic health warnings which will have an effect on smokers who cannot read [73].

These are the first findings at the national level on e-cigarette use in Kenya. Seven respondents reported to have ever used an e-Cigarette. The global estimates of ever use and current use of e-cigarettes are 54.7 and 19.4% among current tobacco smokers and 7.0 and 1.5% among non-smokers respectively [74]. While motivations for usage are not clear, tobacco cessation is associated with increased use of e-cigarettes whether or not users intended to quit smoking cigarettes [75]. In this aspect, the trend is encouraging however future targeted studies need to be done to understand better the drivers of e-cigarette use and to monitor prevalence of usage as it is predicted that e-cigarette use will increase in future [23].

### Policy implications

STEPS data provide the primary data for policy action [76, 77]. The WHO FCTC has established a transformational approach to tobacco control for governments internationally since its inception in 2003 [78]. Kenya is a party to the convention since 2005 and has a comprehensive legislation accommodating most provisions of the treaty. The TCA of 2007 among other things provides for measures to control all forms of tobacco products. It provides for bans on all forms of tobacco advertising, promotion and sponsorship (TAPS), ban on smoking in public places and ban on sales of tobacco products to and by minors. The Act further advocates for tobacco control education, offering of cessation services and raising of tobacco cigarette tax. However, adherence to the TCA by bars and restaurants in Nairobi is not optimal and enforcement needs to be strengthened [49].

Our study demonstrated that men and those with poorer education as well as those that consume alcohol had the most risky tobacco consumption trends. Targeted policy, public health and individual interventions should focus primarily on these vulnerable groups based on results from the study.

Tobacco control is a complex issue requiring a multi-sectoral approach to effectively and comprehensively implement critical policy measures such as those provided for by the FCTC and the TCA. Further, the FCTC and TCA recognizes the key role that the civil society and business establishments could play particularly in advocacy, capacity building and implementation. Public education, including placement of health warnings on tobacco packages, could facilitate rising of public awareness on the harmfulness of tobacco use and exposure to tobacco smoke resulting in behavior change including motivating users to quit and delaying initiation. To address current tobacco use, it is crucial that cessation services including treatment for tobacco dependence are integrated into health service delivery at all levels including at the community. Making such services readily available alongside other population-based measures could help achieve a tobacco-free Kenya.

Additionally, cognizance of the socioeconomic trends which influence the uptake of new and emerging tobacco products could facilitate containment of the tobacco epidemic and therefore have an impact on related morbidity and mortality.

### Limitations

Use of tobacco products is a potentially sensitive question given stigma associated with its usage. Use, frequency and type of tobacco used therefore may have been subject to response bias, however this is the standard for the WHO STEPwise approach [77]. This was likely also not helped by face-to-face interviews that may further bias responses. Additionally, tobacco usage is self-reported and refers to periods preceding the study, subjecting responses to recall bias. Given the cross-sectional nature of the study design, associations can be drawn but causality cannot be assumed. Data related to factors affecting increased tobacco use that are established in the literature such as tobacco use by other family members and peers were not collected in the STEPS survey.

## Conclusion

The use of tobacco has seen concerning trends in the African region in recent decades, Kenya notwithstanding. The 2015 Kenya WHO STEPS was unique in providing primary data on the status quo of tobacco use in the country. Interesting trends by sociodemographic status highlight the importance of focusing on young people, men, and those with lower levels of education. Concurrent usage of alcohol is associated with higher odds of risky tobacco use. Targeting tobacco prevention strategies amongst alcohol users is therefore recommended, including revisiting the public smoke ban for effective implementation. Further data is needed on the use of smokeless tobacco, and its impact on smoked tobacco products, as well as on the novel use of e-cigarettes, with considerations for health and societal implications.

## Abbreviations

DHS: Demographic and Health Surveys
FCTC: Framework Convention on Tobacco Control
GATS: Global Adult Tobacco Survey
LMIC: Low- and Middle-Income Country
NASSEP V: National Bureau of Statistics household-based sampling frame
NCD: Noncommunicable Disease
PDA: Personal Digital Assistant
STEPs: Stepwise approach to surveillance
TAPS: Tobacco Advertising, Promotion and Sponsorship
WHO: World Health Organization

## References

[1] GBD 2015 Risk Factors Collaborators (2016). Global, regional, and national comparative risk assess- ment of 79 behavioural, environmental and occupational, and metabolic risks or clusters of risks, 1990–2015: a systematic analysis for the Global Burden of Disease Study. Lancet.

[2] WHO (2017). WHO report on the global tobacco epidemic, 2017: monitoring tobacco use and prevention policies.

[3] WHO (2014). The WHO framework convention on tobacco control: 10 years of implementation in the african region.

[4] Esamai FO (1998). Relationship between exposure to tobacco smoke and bronchial asthma in children: a review. East Afr Med J.

[5] World Health Organization (2008). WHO Report on the Global Tobacco Epidemic, 2008: The MPOWER Package.

[6] Nturibi EM, Akinsola AK, McCurdy SA (2009). Smoking prevalence and tobacco control measures in Kenya, Uganda, the Gambia and Liberia: a review. Int J Tuberc Lung Dis.

[7] Macigo FG, Mwaniki DL, Guthua SW (1995). The association between oral leukoplakia and use of tobacco, alcohol and khat based on relative risks assessment in Kenya. Eur J Oral Sci.

[8] Owili PO, Muga MA, Pan WC, Kuo HW (2017). Indoor secondhand tobacco smoke and risk of under-five mortality in 23 sub-Saharan Africa countries: a population based study and meta-analysis. PLoS One.

[9] WHO (2015). WHO global report on trends in prevalence of tobacco smoking 2015.

[10] GBD 2015 Tobacco Collaborators (2017). Smoking prevalence and attributable disease burden in 195 countries and territories, 1990–2015: a systematic analysis from the global burden of disease study 2015. Lancet.

[11] Corrao MA, Guindon GE, Cokkinides V, Sharma N (2000). Building the evidence base for global tobacco control. Bull WHO.

[12] Ng M, Freeman MK, Fleming TD, Robinson M, Dwyer-Lindgren L, Thomson B, Wollum A, Sanman E, Wulf S, Lopez AD, Murray CJ, Gakidou E (2014). Smoking prevalence and cigarette consumption in 187 countries, 1980-2012. JAMA.

[13] World Population Prospects: The 2017 Revision. https://population.un.org/wpp/Graphs/Probabilistic/POP/TOT/. Accessed Mar 2018.

[14] Brathwaite R, Addo J, Smeeth L, Lock K (2015). PLoS One.

[15] Sreeramareddy CT, Pradhan PM, Sin S (2014). Prevalence, distribution, and social determinants of tobacco use in 30 sub-Saharan African countries. BMC Med.

[16] Pampel F (2008). Tobacco use in sub-Sahara Africa: estimates from the demographic health surveys. Soc Sci Med.

[17] Peltzer K (2011). Early smoking initiation and associated factors among in-school male and female adolescents in seven African countries. Afr Health Sci.

[18] Veeranki SP, John RM, Ibrahim A, Pillendla D, Thrasher JF, Owusu D, Ouma AE, Mamudu HM (2017). Age of smoking initiation among adolescents in Africa. Int J Public Health.

[19] Ndetei DM, Khasakhala LI, Mutiso V, Ongecha-Owuor FA, Kokonya DA (2009). Patterns of drug abuse in public secondary schools in Kenya. Subst Abus.

[20] Doku D (2010). The tobacco industry tactics – a challenge for tobacco control in low and middle income countries. Afr Health Sci.

[21] Evans SE, Hoffman AC (2014). Electronic cigarettes: abuse liability, topography and subjective effects. Tob Control.

[22] Schroeder M, Hoffman A (2014). Electronic cigarettes and nicotine clinical pharmacology. Tob Control.

[23] Cheng T (2014). Chemical evaluation of electronic cigarettes. Tob Control.

[24] Global Adult Tobacco Survey (GATS). Kenya Report, 2014. http://www.who.int/tobacco/surveillance/survey/gats/Kenya-report-2014.pdf. Accessed 30 May 2018.

[25] Kenya National Bureau of Statistics (2015). Kenya Demographic and Health Survey.

[26] WHO (2015). Global status report on noncommunicable diseases 2014.

[27] Gathecha GK (2014). Tobacco control research in Kenya: the existing body of knowledge. Pan Afr Med J.

[28] Lo TQ, Oeltmann JE, Odhiambo FO, Beynon C, Pevzner E, Cain KP, Laserson KF, Phillips-Howard PA (2013). Alcohol use, drunkenness and tobacco smoking in rural western Kenya. Tropical Med Int Health.

[29] Haregu Tilahun Nigatu, Oti Samuel, Egondi Thaddaeus, Kyobutungi Catherine (2015). Co-occurrence of behavioral risk factors of common non-communicable diseases among urban slum dwellers in Nairobi, Kenya. Global Health Action.

[30] Tobacco Control Regulations 2014. http://kenyalaw.org/kl/fileadmin/pdfdownloads/LegalNotices/169-Tobacco_Control_Regulations__2014. Accessed Mar 2018.

[31] WHO (2017). WHO report on the global tobacco epidemic, 2017. Country profile: Kenya.

[32] WHO Framework Convention on Tobacco Control. http://www.who.int/tobacco/framework/WHO_FCTC_english.pdf. Accessed Mar 2018.

[33] MPOWER Brochures and Other Resources. World Health Organization. http://www.who.int/tobacco/mpower/publications/en/. Accessed Mar 2018.

[34] Perl R, Murukutla N, Occleston J, Bayly M, Lien M, Wakefield M, Mullin S (2015). Responses to antismoking radio and television advertisements among adult smokers and non-smokers across Africa: message-testing results from Senegal, Nigeria and Kenya. Tob Control.

[35] Maina WK, Kitonyo R, Ogwell AE (2013). Using findings from a public opinion poll to build political support for tobacco control policy in Kenya. Tob Control.

[36] Husain MJ, English LM, Ramanandraibe N (2016). An overview of tobacco control and prevention policy status in Africa. Prev Med.

[37] WHO. The WHO STEPwise approach to noncommunicable disease risk factor surveillance (STEPS): WHO STEPS Instrument (Core and Expanded). http://www.who.int/ncds/surveillance/steps/STEPS_Instrument.pdf. Accessed 30 Jan 2018.

[38] Ministry of Health division of noncommunicable diseases (2015). Kenya STEPwise survey for noncommunicable diseases risk factors 2015 report.

[39] Global Adult Tobacco Survey Collaborative Group (2011). Tobacco questions for surveys: a subset of key questions from the global adult tobacco survey (GATS).

[40] Jackson KM, Sher KJ, Cooper ML, Wood PK (2002). Adolescent alcohol and tobacco use: onset, persistence and trajectories of use across two samples. Addiction.

[41] Substance Abuse and Mental Health Services Administration (2013). Results from the 2012 National Survey on drug use and health: summary of National Findings. NSDUH Series H-46.

[42] Britton J (2017). Death, disease, and tobacco. Lancet.

[43] Islami F, Torre LA, Jemal A (2015). Global trends of lung cancer mortality and smoking prevalence. Transl Lung Cancer Res.

[44] Palipudi Krishna M., Gupta Prakash C., Sinha Dhirendra N., Andes Linda J., Asma Samira, McAfee Tim (2012). Social Determinants of Health and Tobacco Use in Thirteen Low and Middle Income Countries: Evidence from Global Adult Tobacco Survey. PLoS ONE.

[45] Ethiopia STEPS Survey 2015 Fact sheet. http://www.who.int/ncds/surveillance/steps/Ethiopia_2015_STEPS_FactSheet.pdf?ua=1. Accessed Mar 2018.

[46] Uganda STEPS Survey 2014 Fact sheet. http://www.who.int/ncds/surveillance/steps/Uganda_2014_STEPS_FactSheet.pdf. Accessed Mar 2018.

[47] Rwanda Non-communicable Diseases Risk Factors Report. http://www.who.int/ncds/surveillance/steps/Rwanda_2012_STEPS_Report.pdf?ua=1. Accessed Mar 2018.

[48] Tanzania STEPS Survey-2012 Fact Sheet. http://www.who.int/ncds/surveillance/steps/UR_Tanzania_FactSheet_2012.pdf?ua=1. Accessed Mar 2018.

[49] Karimi KF, Ayah R, Olewe T (2016). Adherence to the tobacco control act, 2007: presence of a workplace policy on tobacco use in bars and restaurants in Nairobi, Kenya. BMJ Open.

[50] Gichuki JW, Opiyo R, Mugyenyi P, Namusisi K (2015). Healthcare Providers’ level of involvement in provision of smoking cessation interventions in public health facilities in Kenya. J Public Health Afr.

[51] Ndetei DM, Khasakhala LI, Ongecha-Owuor FA, Kuria MW, Mutiso V, Kokonya DA (2009). Prevalence of substance abuse among patients in general medical facilities in Kenya. Subst Abus.

[52] Naanyu V, Asirwa CF, Wachira J, Busakhala N, Kisuya J, Otieno G, Keter A, Mwangi A, Omenge OE, Inui T (2015). Lay perceptions of breast cancer in Western Kenya. World J Clin Oncol.

[53] Bilano V, Gilmour S, Moffiet T (2015). Global trends and projections for tobacco use, 1990–2025: an analysis of smoking indicators from the WHO Comprehensive information Systems for Tobacco Control. Lancet.

[54] Baleta A (2010). Africa’s struggle to be smoke free. Lancet.

[55] Sreeramareddy CT, Pradhan PM (2015). Prevalence and social determinants of smoking in 15 countries from North Africa, central and Western Asia, Latin America and Caribbean: secondary data analyses of demographic and health surveys. PLoS One.

[56] Seltzer CC (1959). Masculinity and smoking. Science.

[57] Starr ME (1984). The Marlboro man: cigarette smoking and masculinity in America. Pop Cult.

[58] Pachankis JE, Westmaas JL, Dougherty LR (2011). The influence of sexual orientation and masculinity on young men’s tobacco smoking. J Consult Clin Psychol.

[59] Hunt K, Hannah MK, West P (2004). Contextualizing smoking: masculinity, femininity and class differences in smoking in men and women from three generations in the west of Scotland. Health Educ Res.

[60] Amos A, Greaves L, Nichter M, Bloch M (2012). Women and tobacco: a call for including gender in tobacco control research, policy and practice. Tob Control.

[61] Allen AM, Oncken C, Hatsukami D (2014). Women and smoking: the effect of gender on the epidemiology, health effects, and cessation of smoking. Curr Addict Rep.

[62] Torchalla I, Okoli CT, Bottorff JL, Qu A, Poole N, Greaves L (2012). Smoking cessation programs targeted to women: a systematic review. Women Health.

[63] Bottorff JL, Haines-Saah R, Kelly MT, Oliffe JL, Torchalla I, Poole N, Greaves L, Robinson CA, Ensom MH, Okoli CT, Phillips JC (2014). Gender, smoking and tobacco reduction and cessation: a scoping review. Int J Equity Health.

[64] Africa WROf (2014). Towards tobacco-free young people in the African region.

[65] Preventing a tobacco epidemic in Africa. A call for effective action to support health, social, and economic development. Report of the committee on the negative effects of tobacco on Africa’s health, economy, and development. http://www.nationalacademies.org/asadi/Africa tobacco control-FINAL.Pdf. Accessed Mar 2018.

[66] World Health Organisation Regional Office for Africa. Facts on tobacco use in the Africa Region. http://www.afro.who.int/sites/default/files/2017-06/facts-on-tobacco-use-in-the-african-region.pdf. Accessed Mar 2018.

[67] DHHS Report of the Surgeon General. The health consequences of smoking—50 years of Progress. https://www.surgeongeneral.gov/library/reports/50-years-of-progress/index.html. Accessed Mar 2018.

[68] Concurrent Alcohol and Tobacco Dependence [Internet]. https://pubs.niaaa.nih.gov/publications/arh26-2/136-142.htm. Accessed Mar 2018.

[69] Verplaetse TL, McKee SA (2017). An overview of alcohol and tobacco/nicotine interactions in the human laboratory. Am J Drug Alcohol Abuse.

[70] Kalman D, Kim S, DiGirolamo G, Smelson D, Ziedonis D (2010). Addressing tobacco use disorder in smokers in early remission from alcohol dependence: the case for integrating smoking cessation services in substance use disorder treatment programs. Clin Psychol Rev.

[71] West R, Raw M, McNeill A, Stead L, Aveyard P, Bitton J, Stapleton J, McRobbie H, Pokhrel S, Lester-George A, Borland R (2015). Health-care interventions to promote and assist tobacco cessation: a review of efficacy, effectiveness and affordability for use in national guideline development. Addiction.

[72] Skov-Ettrup LS, Ringgaard LW, Dalum P, Flensborg-Madsen T, Thygesen LC, Tolstrup JS (2014). Comparing tailored and untailored text messages for smoking cessation: a randomized controlled trial among adolescent and young adult smokers. Health Educ Res.

[73] Republic of Kenya. Tobacco Control Regulations 2014. http://kenyalaw.org/kl/fileadmin/pdfdownloads/LegalNotices/169-Tobacco_Control_Regulations__2014. Accessed Aug 2017.

[74] Hennessy EA, Tanner-Smith EE, Steinka-Fry KT (2015). Do brief alcohol interventions reduce tobacco use among adolescents and young adults? A systematic review and meta-analysis. J Behav Med.

[75] Caponnetto P, Campagna D, Cibella F, Morjaria JB, Caruso M, Russo C, Polosa R (2013). EffiCiency and safety of an eLectronic cigAreTte (ECLAT) as tobacco cigarettes substitute: a prospective 12-month randomized control design study. PLoS One.

[76] Golechha M (2016). Health promotion methods for smoking prevention and cessation: a comprehensive review of effectiveness and the way forward. Int J Prev Med.

[77] Riley L, Guthold R, Cowan M (2016). The World Health Organization STEPwise approach to noncommunicable disease risk-factor surveillance: methods, challenges, and opportunities. Am J Public Health.

[78] WHO report on the Global epidemic 2015. Raising taxes on tobacco. http://apps.who.int/iris/bitstream/handle/10665/178574/9789240694606_eng.pdf?sequence=1. Accessed Mar 2018.

